# Uremic Sarcopenia: Clinical Evidence and Basic Experimental Approach

**DOI:** 10.3390/nu12061814

**Published:** 2020-06-18

**Authors:** Hiroshi Nishi, Koji Takemura, Takaaki Higashihara, Reiko Inagi

**Affiliations:** 1 Division of Nephrology and Endocrinology, The University of Tokyo Graduate School of Medicine, 7-3-1 Hongo, Bunkyo-ku, Tokyo 113-8655, Japan; ktakemura@hotmail.co.jp (K.T.); fyqfj205@gmail.com (T.H.); 2 Division of CKD Pathophysiology, The University of Tokyo Graduate School of Medicine, 7-3-1 Hongo, Bunkyo-ku, Tokyo 113-8655, Japan

**Keywords:** kidney, sarcopenia, skeletal muscle, uremia

## Abstract

Sustained physical activity extends healthy life years while a lower activity due to sarcopenia can reduce them. Sarcopenia is defined as a decrease in skeletal muscle mass and strength due not only to aging, but also from a variety of debilitating chronic illnesses such as cancer and heart failure. Patients with chronic kidney disease (CKD), who tend to be cachexic and in frail health, may develop uremic sarcopenia or uremic myopathy due to an imbalance between muscle protein synthesis and catabolism. Here, we review clinical evidence indicating reduced physical activity as renal function deteriorates and explore evidence-supported therapeutic options focusing on nutrition and physical training. In addition, although sarcopenia is a clinical concept and difficult to recapitulate in basic research, several in vivo approaches have been attempted, such as rodent subtotal nephrectomy representing both renal dysfunction and muscle weakness. This review highlights molecular mechanisms and promising interventions for uremic sarcopenia that were revealed through basic research. Extensive study is still needed to cast light on the many aspects of locomotive organ impairments in CKD and explore the ways that diet and exercise therapies can improve both outcomes and quality of life at every level.

## 1. Uremic Sarcopenia as a Clinical Entity

“Sarcopenia” is derived from the Greek word for “muscle loss” as a combination of “sarx” for meat and muscle and “penia” for loss [1]. This concept was originally proposed by Rosenberg [2] and denotes a syndrome characterized by a progressive and systemic decrease in muscle mass and strength, leading to physical dysfunction, poor quality of life (QOL), and the risk of death. Age-related muscle loss is classified as primary sarcopenia, while that associated with a physical handicap or chronic illness is classified as secondary sarcopenia. The diagnostic criteria of the European Working Group on Sarcopenia in Older People (EWGSOP) are used globally [3], while, considering the differences in physique between races or ethnicities, for instance, the criteria developed by the Asian Working Group for Sarcopenia (AWGS) are recommended for Asians [4]. Many measures of skeletal muscle mass are quantitated with a cut-off value from the dual-energy X-ray absorptiometry (DEXA) method, while others adopt a cut-off value using the bioelectrical impedance analysis (BIA) method with a mathematical equation based on upper arm circumference and subcutaneous fat, in conjunction with a subjective global assessment.

Chronic kidney disease (CKD) is one of the chronic illnesses which complicates sarcopenia [5]. According to NHANES III (1994–1998), muscle mass is frequently reduced in CKD patients who have a reduction in glomerular filtration rate (GFR) or progression of albuminuria [6]. Another cohort study illustrated that walking speed and muscular strength deteriorated in those with 60–89 mL/min/1.73 m^2^ and below 60 mL/min/1.73 m^2^, compared to those with a creatinine clearance of 90 mL/min/1.73 m^2^ or more [7]. According to an observational study analyzing pre-dialysis CKD patients who were provided only minimal dietary counseling by health professionals, lower GFR was significantly associated with a spontaneous lower protein intake [8]. The authors warranted that dietary protein restriction should be carefully introduced for advanced CKD patients because their spontaneous protein intake is already low. According to the Korea National Health and Nutrition Examination Survey (KNHANES), the frequency of muscle loss decreases as the stage of CKD progresses, showing a 2.6%, 5.6%, 18.1% decrease in frequency in male G1 (estimated GFR >90 mL/min/1.73 m^2^), G2 (60 to 90 mL/min/1.73 m^2^), and G3 to G5 (<60 mL/min/1.73 m^2^), respectively [9]. Collectively, however, sarcopenia prevalence is greater in CKD patients than in the general population and increases with CKD advancement.

Unfortunately, the prognosis of CKD patients with sarcopenia is not promising in terms of mortality and length of hospital stay. In a report examining urinary creatinine excretion as an indicator of skeletal muscle mass in a body, the risk of death increased as urinary creatinine excretion decreased [10]. In CKD stages G3 to G5 diagnosed with sarcopenia based on the BIA method, muscular complication is associated with a poor prognosis [11]. Additionally, CKD patients with reduced physical functions, such as walking speed and grip strength, have a worse prognosis than those with CKD where those functions are retained [12]. According to epidemiological studies on end-stage renal disease, the incidence of sarcopenia increases as renal function deteriorates [13] and the incidence in elderly hemodialysis (HD) patients is significantly heightened [14]. The importance of uremic sarcopenia lies in its impact on mortality and morbidity, including susceptibility to fractures that affect the QOL for CKD patients [15], cardiovascular events [16], and overall survival [17].

Epidemiological studies have illustrated that quite a few factors may contribute to the increased incidence of sarcopenia in CKD patients, including limited protein intake, energy deficiency, aging, insufficient or deficient exercise, inflammation, metabolic acidosis, lack of natural vitamin D, and even diuretic treatment [18,19,20]. Additionally, abnormal lipid metabolism and obesity are frequently associated with sarcopenia in advanced CKD patients [21]. Truncal deposit of fat is also related to high blood levels of hepatic growth factor (HGF). HGF serves as a marker for obesity in the general population, and is predictive of mortality in catabolic conditions [22,23]. Similarly, the concept of sarcopenia obesity attracts attention as a condition that has both skeletal muscle loss and increased body fat, and, however, it has been reported that sarcopenia obesity is not a risk for mortality in CKD patients [24]. In the United States, obesity is high in both the general population and CKD patients, and the study may include sarcopenia obesity.

Uremic myopathy, a term similar to uremic sarcopenia is a defined skeletal complication in CKD patients [25,26]. Both are associated with a variety of muscular symptoms including weakness or atrophy in uremic patients, although uremic myopathy may focus on the disease per se regardless of any local or systemic impairment. Sarcopenia implies more systemic and dysfunctional disorders that are also defined by international guidelines. However, uremic sarcopenia and uremic myopathy can be hard to distinguish, particularly in laboratory research.

## 2. Nutritional Therapy for Uremic Sarcopenia: The Protein Intake Dilemma

Protein intake adjustment and energy levels play a key role in diet therapy for CKD. Several meta-analyses concluded that a low protein diet is effective in reducing composite endpoints including mortality in nondiabetic CKD patients [27,28]. The Modification of Diet in Renal Disease (MDRD) study provided significant evidence more consistent with the hypothesis of a beneficial effect of protein restriction [29,30]. One recent major review maintains that it is the patient’s preference to adopt dietary interventions for the conservative management of uremia [31]. Notably, a small number of RCTs have been reported, in which strict protein restriction (<0.6 g/kg/day), in combination with a keto acid supplement, was able to postpone ESRD and reduce the rate of renal function decline in advanced CKD [32,33]. Unfortunately, not all CKD patients may be capable of undertaking this strict approach for an extended period of time.

For metabolically stable adults with CKD, the National Kidney Foundation-Kidney Disease Outcomes Quality Initiative (NKF-KDOQI) plans to recommend prescribing an energy intake of 25 to 35 kcal/kg lean body mass (LBM) per day based on age, gender, level of physical activity, body composition, weight status goals, CKD stage, and concurrent illness or presence of inflammation to maintain normal nutritional requirements [34]. As for protein intake, in metabolically stable adults with CKD 3–5, they recommend protein restriction supervised by a registered dietitian nutritionist (RDN) or equivalent in collaboration with a physician, suggesting a low protein diet providing 0.55 to 0.60 g dietary protein/kg ideal body weight/day, or a very-low protein diet providing 0.28 to 0.43 g dietary protein/kg ideal body weight (IBW)/day with additional keto acid analogs to meet protein requirements (0.55 to 0.60 g/kg body weight/day). For metabolically stable adults with CKD on maintenance hemodialysis (MHD), they recommend prescribing a dietary protein intake of 1.0 to 1.2 g/kg ideal body weight per day. Having diabetes is not regarded as metabolically stable, so for the adults with CKD who have diabetes, NKF-KDOQI presents the option of prescribing a greater amount of dietary protein intake of 0.8 to 0.9 g/kg of ideal body weight per day.

Sarcopenia may present a more complex status in terms of metabolic stability, as skeletal protein catabolism is predominant in protein synthesis and degradation. For elderly people without CKD, adequate protein intake of 1.0 to 1.2 g/kg/day is considered effective for the prevention and improvement of sarcopenia [35], which is clearly incompatible with the protein restriction mentioned as a diet recommended for CKD patients. Thus, dietary therapy for uremic sarcopenia has not been well established. Strict adherence to any nutritional guidance on protein restriction is inappropriate and the disease activity, risk, and compliance of individual patients should be taken into consideration. An individual approach is essential to evaluate the risks of both CKD and sarcopenia and to select an appropriate protein intake for each patient.

## 3. Physical Exercise to Prevent or Regress Uremic Sarcopenia

Exercise therapy is designated to enhance or maintain muscle strength, or at least to halt the progression of muscular function loss, which is expected to play a beneficial role in uremic sarcopenia. In addition to mortality or renal prognosis, as endpoints that pertain to physical activity, cardiopulmonary function, walking capacity, and skeletal muscle strength and mass are evaluated. In a cardiopulmonary exercise test, VO_2_ peak or VO_2_ max (representing the maximum rate of oxygen consumption measured during incremental exercise) is the most frequently monitored. For evaluation of walking capacity, the six minute walk test and walking speed measurement are frequently adopted. Muscular strength is measured mainly with a handheld dynamometer, although manual muscle testing and other types of dynamometry are conventionally available. Additionally, physical activity is assessed as a part of the QOL evaluation with the Kidney Disease Quality of Life instrument (KDQOL) [36].

For aging sarcopenia, multiple meta-analyses showed no obvious effect of diet alone on improving skeletal muscle mass, physical function, and muscle strength and, therefore, nutrition should be combined with physical exercise therapy when treating sarcopenia [37,38,39,40,41,42,43]. For uremic sarcopenia, a series of small size cohort studies analyzed the exercise effect on mortality, renal prognosis, physical activity, and QOL in patients with various stages of CKD (Table 1).

**Figure 1:**
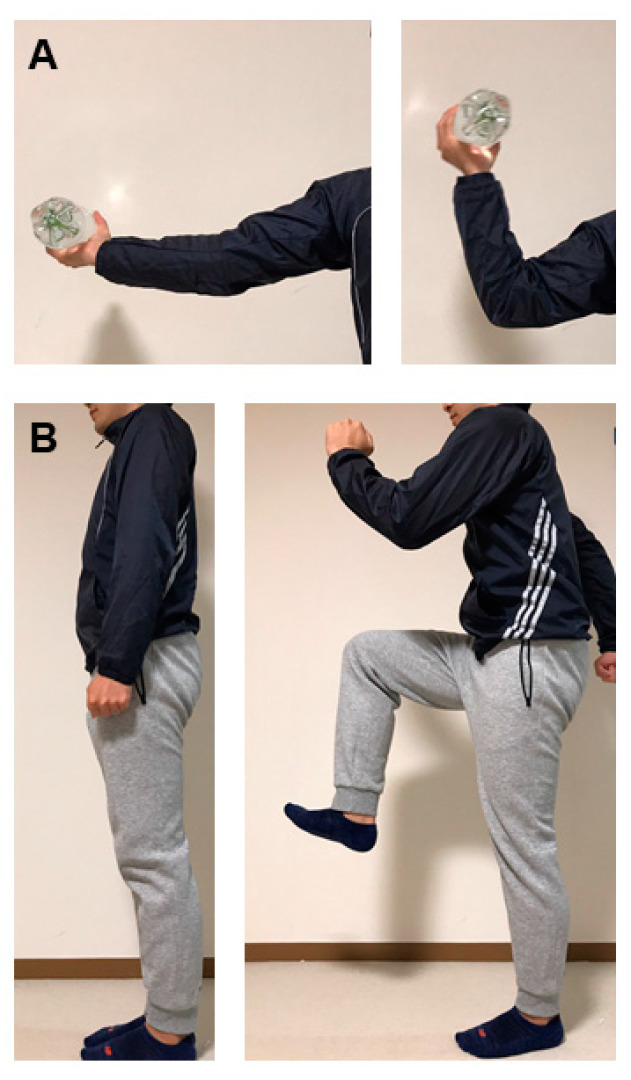
Suggested exercise therapy intervention for patients with uremic sarcopenia. (**A**) Biceps curl as an example of resistance training. A person holds one bottle with each hand down, raises it until it reaches his or her shoulders’ height and slowly lowers them back down after a short pause. A plastic bottle filled with water as a dumbbell can be used. (**B**) Walking in place as an example of aerobic exercise. A person lifts his or her knee up until the calf and thigh form a right angle, then, lowers that leg and repeats with the other leg. The above steps are repeated 10 times. This movement combines traditional running movements with knee lifting.

In most clinical trials, physical exercise therapy is implemented as resistance training, aerobic exercise, or both (Figure 1). While resistance exercise consists of a moving the target musclesby squeezing handgrips and raising dumbbells repeatedly, the aerobic exercise involves stomping and slowly raising and lowering the legs. The training program should be systemic with plans to progressively address the CKD patients’ weaknesses and to maximally condition the patients for participation in regular activities. Since CKD patients are usually elderly, supervised and personalized exercise is highly recommended.

### 3.1. Exercise for Patients with Pre-Dialysis CKD

A recent study with pre-dialysis CKD patients revealed that home-based aerobic and resistance exercises increased grip strength and knee extension muscle strength [44]. Self-administered exercise training programs were shown to improve handgrip and isometric quadriceps strength [45]. Another study of patients with CKD stage G3b to G4 indicates that muscle anatomical cross-sectional area, muscle volume, and knee extensor strength are increased by a supervised progressive resistance exercise program, although recruitment rates were low [46]. Moreover, several studies demonstrated that exercise therapy that consisted of aerobatic exercise, intermittent exercise, and resistance training, improved VO_2_ peak [48,49,50,51]. Other reports showed that the exercise therapy heightened six minute walk test scoring [52,53]. Physical functioning and vitality in QOL parameters also improved in several studies [52,54].

Multiple studies showed that the deterioration of renal function in patients with pre-dialysis CKD slowed down after lifestyle changes including exercise and diet modification [55]. Therefore, exercise therapy combined with appropriate diet therapy is beneficial to suppress CKD progression, particularly in obese and diabetic patients, although the long-term effect on CKD progression from exercise therapy alone remains unknown.

Although amelioration of uremic sarcopenia aims at reducing mortality or hospitalization, very few clinical studies have evaluated death or hospitalization as an outcome for pre-dialysis CKD patients who underwent exercise therapy. In one study that assesses the clinical effect of 6 years of lifestyle intervention in persons with impaired glucose tolerance developing microvascular complications over a 20 year period, renal replacement therapy and mortality did not decrease as a result of interventions that included exercise therapy [56].

Therefore, exercise therapy may be beneficial for maintaining or improving skeletal muscle performance and exercise endurance in pre-dialysis CKD patients.

### 3.2. Exercise for Patients Undergoing Hemodialysis

In comparison to pre-dialysis CKD and kidney transplant patients, patients on dialysis therapy are more extensively analyzed in terms of the effects of exercise therapy.

For patients on hemodialysis, it is controversial whether exercise could increase skeletal muscle mass [76,77,78]. Exercise-induced enhancement of skeletal muscle strength is shown by several human studies [47,57,58], but not supported by others [68,79,80]. Although clinical evidence of the effect of exercise therapy on mortality in hemodialysis patients is limited, multiple studies support that VO_2_ peak [59,60,61,62,63,64] or VO_2_ max [65,66] was increased by exercise intervention, which is better supported by clinical studies with an observation period of six months or longer [81]. In one report, walking capacity evaluated in a six minute walk test was improved as the result of exercise therapy [67]. In addition, several studies pointed out that physical activity components of the QOL assessment were enhanced [68,69,70].

Therefore, exercise therapy may help improve exercise endurance, which is supported by a more significant number of clinical studies targeting hemodialysis patients, compared to those targeting pre-dialysis patients. Of note, few clinical studies analyzed uremic sarcopenia in peritoneal dialysis patients [82].

### 3.3. Exercise for Kidney Transplant Recipients

A recent single-blind, randomized controlled trial with adult kidney transplant recipients showed that intensive nutrition intervention including exercise counseling failed to improve physical functioning, such as gait speed, sit to stand, and grip strength, although exercise intervention was limited, i.e., 3 exercise physiologist visits over 12 months [71]. One report with a small sample size of renal transplant recipients illustrated that VO_2_ peak is increased by exercise [72]. Several reports showed that physical activity components in QOL are improved by exercise [72,73]. However, exercise therapy is not shown beneficial for preserving or improving the graft function in kidney transplant recipients [74,75].

## 4. Experimental Laboratory Tools for Exploration of Uremic Sarcopenia Mechanism

**Figure 2:**
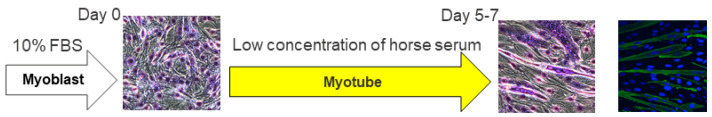
General protocol for mouse skeletal C2C12 myoblast differentiation to myotubes. The immortal line of mouse skeletal myoblasts is kept in culture, avoiding high confluence culture conditions. Replacement of C2C12 myoblast culture medium with growth/differentiation medium containing the lower concentration of horse serum for five to seven days promoted differentiation to C2C12 myotube, which are fused with each other and represent a polynuclear syncytial morphology, as shown with Giemsa staining (left and middle panels) and immunofluorescence staining with antibody against myosin heavy chain and Höchst 33,258 (right panel). FBS: fetal bovine serum.

A number of nutritional and physical exercise approaches to uremic sarcopenia have been proposed, but these are mostly investigated in the preclinical stage. This underlines the importance of basic research which focuses on uremic sarcopenia. In biological experiments on skeletal muscle, culture myocyte lineage such as mouse C2C12 cells [83,84] are widely used in addition to rat L6 cells and skeletal muscle cells (SkMC) established from various animal species such as human, rat, and mouse [85]. C2C12 cells, subcloned from normal adult C3H mouse leg muscle, are widely used to study in vitro myogenesis and cell differentiation (Figure 2). Primary cultured cells also are available by growing satellite cells (myoblast) isolated from the animal muscle tissue, although skillful technique is required [86]. For experimental studies analyzing physiological aspects of skeletal muscle such as the insulin-dependent glucose uptake function, isolation and culture of epitrochlearis muscles in a medium consisting of Krebs Ringer bicarbonate (KRB) buffer, with sodium pyruvate in the presence of insulin, is another established protocol [87]. To mimic uremic sarcopenia in vitro, chemical compounds known as uremic toxins or uremic serum sampled from CKD animals are added to the culture media.

Obviously, since uremic sarcopenia is a clinical entity and concept in humans, appropriate animal models should be established. A variety of rodent models have been employed so far to recapitulate human uremic sarcopenia. This review will focus on rodent models designed to mimic uremic sarcopenia (Table 2). However, they have limitations in their characterization and in the reproducibility of the condition [88]. The search for novel models is still warranted. Due to the limited availability and very high costs of using older animals, the use of young animals could be another drawback as a majority of uremic patients with sarcopenia are elderly, complicated by primary, aging sarcopenia.

### 4.1. Subtotal (5/6) Nephrectomy

Subtotal nephrectomy, also known as 5/6 nephrectomy, is probably the most established method to mimic progressive renal failure showcasing renal glomerulosclerosis, interstitial fibrosis, and systemic hypertension, resembling a mass reduction of the kidney in humans. 129/Sv and Swiss-Webster mice can develop more severe damage in terms of renal function and histology compared to C57BL/6 mice. Two different protocols, the ligation model and the ablation model, have been widely adopted. In both models, in order to lighten the burden imposed on an animal, whole procedures are performed in two stages. In approximately two weeks, renal function is deteriorated as shown by the increased urea nitrogen and creatinine in the blood. Sham mice underwent surgery without damaging the kidneys [89,111].

The ligation model is more suitable for rats rather than mice as careful surgical technique is required [112]. After one kidney is removed (contralateral uninephrectomy), typically, 2/3 branches of the renal artery in the rat are ligated, resulting in an obstruction of blood flow to the upper and lower poles of the remaining kidney. Again, this approach cannot be performed in mice due to their limited renal artery branching. The ablation model can be achieved in both rats and mice. At one to two weeks after uninephrectomy, 50–70% of the remaining kidney is removed by polar excision with electrical knife ablation, for instance.

Regarding skeletal muscle manifestation, several groups observed skeletal muscle atrophy presenting with not only bodyweight reduction, but also with a decrease of the total mass and CSA of the muscle compared with sham mice [89,90,91,92,93,94,95,96,97,98,99,100,101,102]. Treadmill running distance [93,113] and hanging time on the grid with four limbs [102] were shortened and grip strength had also deteriorated [89], while some parameters indicating lowered physical activity were not consistently affected [94,113]. In addition, mouse ambulation, as indicated by overnight cage activity and swimming velocity, was reduced [90]. Histologically, skeletal fibrosis was noted [95] and the number of CD31-positive capillaries was decreased [102]. In terms of molecular biology, elevation of myostatin, atrogin-1, muscle RING-finger protein-1 (MuRF-1), and an increase in an inflammatory signal such as IL-6, and acceleration of muscle protein degradation are also noted [91,94,96,99], although alteration of muscular protein synthesis varies between reports.

Extensive research casts light on metabolic aspects targeting the skeletal muscle of this uremic model. For example, the mitochondrial amount measured as the ratio of the mitochondrial DNA to nuclear DNA was decreased in the uremic skeletal muscle [113]. Peroxisome proliferator-activated receptor gamma coactivator 1-alpha (PGC-1α) is a transcriptional coactivator that regulates the genes involved in energy metabolism. In subtotal nephrectomized mice, the PGC-1α expression level was decreased in the skeletal muscle, associated with an increase in the methylation ratio of the cytosine residue at 260 base pairs upstream of the initiation point [98,114]. Additionally, 5’-AMP-activated protein kinase (AMPK) has a role as an energy sensor in peripheral tissues and promotes catabolism during energy deficiency. In skeletal muscle, activation of AMPK promotes fatty acid uptake into the mitochondria and subsequent fatty acid oxidation. In subtotal nephrectomized mice, despite a high AMP:ATP ratio, AMPK activation is suppressed not only in the kidney but also in the skeletal muscle [101]. Autophagy-related molecule expression is also increased in those animal muscles, including Bnip3, Beclin-1, and LC3II mRNAs, indicating that autophagosome–lysosome formation is accelerated in uremic skeletal tissue [98,99]. This fused structure forms autolysosomes, in which the sequestered cargos are degraded and recycled for the maintenance of cellular homeostasis [115,116]. Thus, it may promote skeletal muscle wasting in CKD [98,117,118].

### 4.2. Adenine Diet Administration

Adenine, also called vitamin B4, is a nucleobase of a purine derivative that is known to be toxic to the kidney. The adenine-induced uremia animal model has gained attention due to its relative ease of implementation without surgery, such as vessel ligation or partial organ ablation. Orally administered adenine is metabolized to 2,8-dihydroxyadenine, which forms crystals in the proximal tubules conferring tubulointerstitial inflammation and fibrosis [119]. In rodents, a number of studies have modified the concentration and duration of adenine in feed based on what was originally developed [120]. The male mice are more vulnerable to renal dysfunction than the female mice [121]. However, regardless of the sex, even with a mixture of adenine with chow, food intake is reduced [122].

With respect to the uremic effect on skeletal muscle, six to seven week 0.2% (*w*/*w*) adenine-fed mice display skeletal muscle atrophy [23,103]. Detailed analysis with liquid chromatography-tandem mass spectrometry revealed the accumulation of uremic toxins and enhanced expression of inflammatory genes in the skeletal muscle [23]. Interestingly, when an obese “pound” mouse that is deficient in functional leptin receptor isoforms is fed with oral adenine (approximately 4.5 mg/day), kidney damage was worse, representing greater tubular injury with a decrease in kidney mitochondria, a lower tissue ATP level, and enhanced oxidative stress [104]. Even pound mice with levels of renal function similar to those of adenine-treated wild-type mice also developed muscle atrophy along with lower tissue ATP and phosphate levels. This worsening of the sarcopenic phenotype in adenine-administered pound mice may mimic obesity sarcopenia in the real clinical setting [24].

### 4.3. Indoxyl Sulfate Administration

Uremic toxin consists of a group of molecules that are usually filtered and excreted by healthy kidneys but are accumulated in the body when renal clearance is impaired. Those compounds are considered directly harmful to various tissues and organs which have been extensively studied, particularly in the context of uremic cardiovascular disease and kidney damage [123].

Accumulation of uremic toxins is reported to trigger skeletal muscle loss or dysfunction in CKD [103,105,124]. Therefore, given that indoxyl sulfate (IS) is a major offender in skeletal muscle loss, independent of another uremic milieu, IS administration is sufficient to induce sarcopenia. In an animal with normal renal function, IS is immediately excreted into the urine despite a slightly elevated level of IS concentration. Therefore, to expect IS accumulation in the body, renal clearance would be reduced. At one week after a unilateral kidney was surgically excised, mice were intraperitoneally administrated IS (100 mg/kg/day) for 12 weeks [105]. This method results in the reduction in weight of the body and tibias anterior (TA), soleus, and gastrocnemius (GC) muscles, despite no significant change in serum creatinine and urea levels. The skeletal manifestation develops simultaneously with an increased expression of myostatin, atrogin-1, IL-6, and TNF-α. Additionally, this type of uremic toxin-mediated skeletal muscle atrophy may target predominantly fast-twitch fibers (Higashihara, Nishi, unpublished). This model is superior in that the IS effect on muscle atrophy can be evaluated.

### 4.4. Cy/+ Rat, a Han:SPRD Rat with Polycystic Kidney

In rodents with uremic sarcopenia, kidney and muscle dysfunction are established within a few weeks or months, while CKD and sarcopenia progress year by year in humans. So, a test subject with slowly progressive renal dysfunction complicated by muscle atrophy would be appropriate for the analysis of uremic sarcopenia. For that reason, Cy/+ rat, a Han:SPRD rat with autosomal dominant polycystic kidney disease (ADPKD) is appropriate, as their kidney function deteriorates month by month. The male Cy/+ rat, a Han:SPRD rat with autosomal dominant polycystic kidney disease, develops persistent azotemia, starting at about 10 weeks of age, which progresses to uremia by about 40 weeks [125].

Despite no difference in weight, the CSA, and fiber-type proportions of muscle, ADPKD rats demonstrate lower maximum torque in ankle dorsiflexion and shorter time to maximum torque, and longer half relaxation time in dorsiflexion and plantarflexion when the right hind limb is electrically stimulated under general anesthesia [106]. These rats also express disrupted sarcomeres, engorged muscle mitochondria, and a higher level of proteolytic markers such as atrogin-1 and MuRF-1 in the skeletal muscle and 8OHdG, an oxidative stress marker, in the bloodstream [107].

### 4.5. Diabetic Kidney Injury

Hyperglycemia is a risk factor for muscle mass and functional reduction [126] and diabetic kidney disease (DKD) is a worldwide public health problem, although the terminology is under discussion. A decreased regenerative capacity after trauma could be a hallmark, in skeletal muscle, of one of the factors contributing to the pathogenesis of sarcopenia [127,128]. As an experimental approach in skeletal muscle biology, skeletal muscle regeneration induced by local injection of a toxic chemical such as cardiotoxin is widely used [129,130].

In a type 1 diabetes mouse intraperitoneally injected with streptozotocin (STZ, 150 mg/kg) for two consecutive days, skeletal muscles obtained 12 weeks after the second STZ injection were atrophic, representing smaller CSA of muscle fibers and weakened grip together with elevated expression of myostatin, atrogin-1, and MuRf-1 in the muscle [108]. In addition, in KKAy mice mimicking type 2 diabetes, obese diabetic db/db mice, fat was ectopically accumulated in the lower limb and the deposition was enhanced after local injection of cardiotoxin [109]. Since type 1 diabetic STZ-treated mice lack this phenotype, insulin resistance may be involved in sarcopenic obesity [109].

### 4.6. Kidney Ischemia-Reperfusion Injury

Renal ischemia-reperfusion by temporarily occluding the renal pedicle(s) with nontraumatic clips is an established rodent model that leads to acute kidney injury (AKI) [131,132], and thus frequent and major kidney disease with high mortality in clinical settings. To sustain the renal damage, bilateral ischemia-reperfusion injury (IRI) [133], a unilateral IRI after uninephrectomy [134], and a unilateral IRI preceding uninephrectomy [135] are introduced.

Concerning muscle function, bilateral IRI induced by an hour occlusion confers poor motor coordination and balance in the rotarod experiment after one week, while grip strength was not altered [110]. Deterioration in the rotarod test indicates a deficit in motor coordination or fatigue resistance, so this could be worth investigating, as behavioral deficits occur even with mild renal failure, which may be due to uremic encephalopathy rather than uremic sarcopenia [136].

## 5. Exploration of Interventions to Overcome Uremic Sarcopenia

As a therapeutic approach to uremic sarcopenia, the muscle protective potentials are reviewed with a focus on basic research in vivo. Some of these approaches simultaneously alleviate the kidney injury so that direct influence on skeletal muscles are indistinguishable unless a conditional genetic ablation system is adopted.

### 5.1. Resistance Training

As a human clinical trial shows, resistance exercise training is capable of preserving lean body mass, nutritional status, and muscle function in patients with moderate chronic kidney disease [137]. Of note, this effect was confirmed in participants consuming a low-protein diet, but still is a promising approach for uremic sarcopenia in subtotal nephrectomized mice. However, since forcing experimental animals to undergo resistance training is unrealistic, several devices to overcome this hardship have been employed. By removing GC and soleus muscles from both hind limbs to overload plantaris muscles, resistance exercise on the muscle can be simulated. Compared to endurance training (treadmill running), resistance training effectively corrected protein synthesis and levels of mediators of protein synthesis such as phosphorylated mTOR and p70S6K, and showed an increase in muscle progenitor cell number and activity [91].

Moreover, acupuncture plus low-frequency electrical stimulation is focused on as a treatment to replicate the benefits of resistance exercise through stimulation of muscle contraction. Enforced muscle contraction by this electrical stimulation works to prevent muscle atrophy [94] and demonstrates autophagy as the promising molecular culprit [98]. This is interesting in that core autophagy proteins are bound to the endoplasmic reticulum (ER) by the organelle membrane-localized cargo receptors and that ER stress is known to target the kidney [138,139,140] as well as uremic skeletal muscle [141].

Aquaporin-4 (AQP4) is a water channel expressed at the sarcolemma of fast-twitch skeletal muscle fibers and possibly modulates homeostasis in myofibers through the regulation of water transport and osmotic pressure [142,143]. Intriguingly, physical exercise modulates AQP4 expression at the post-translational level in muscles of rodents [144], whereas AQP4 gene expression is decreased in the aged rodents [145]. These findings could pinpoint the transporter protein as a key molecule to underlie uremic sarcopenia.

### 5.2. Mitochondrial Metabolism Activation

There is growing evidence that the skeletal muscle of patients in advanced CKD stages is characterized by mitochondrial dysfunction [101,103,146]. Analysis of human muscle biopsy samples pinpointed lower mitochondrial volume density, lower mitochondrial DNA copy number, and higher BNIP3 content [146]. Culture experiments of C2C12 myocytes with uremic toxin revealed the upregulation of glycolysis with excess antioxidative response leading to mitochondrial dysfunction and ATP shortage [103]. Pyruvate dehydrogenase complex converts pyruvate into acetyl-CoA by pyruvate decarboxylation, and acetyl-CoA may then be used in the citric acid cycle to carry out mitochondrial cellular respiration. Miyashita’s group elegantly revealed that activation of pyruvate dehydrogenase by dichloroacetate effectively recovered the treadmill running distance of subtotal nephrectomized mice, which was interestingly decreased by dietary protein [93]. The same lab reported that a diet containing 0.03% (*w*/*w*) 5-aminolevulinic acid (ALA), serving as electron carriers in the mitochondrial electron transport system, increases both skeletal muscle weight and the mitochondrial amount and enhances grip strength and treadmill endurance [113]. Thus, activation of muscle mitochondria metabolism would serve as a potential strategy for combatting uremic sarcopenia.

### 5.3. Myostatin Inhibition

Myostatin is a molecule that suppresses skeletal muscle growth as is dramatically illustrated in myostatin deficient cattle [147]. Myostatin, also called growth differentiation factor-8 (GDF-8), is a member of the TGF-beta family. The N-terminal peptide is secreted from skeletal muscle and is transformed to a mature form when cleaved by metalloprotease. Myostatin binds to the activin receptor type IIB that has serine/threonine kinase activity, and the signals are transduced through two cascades, Smad2/3 and p38MAPK. Smad2/3 is a transcription factor that not only binds directly to DNA to induce transcription of target genes but also directly binds to Forkhead box O (FoxO) to control the ubiquitin E3 ligases atrogin-1 and MuRF-1, resulting in ubiquitin-proteasome-mediated protein degradation [148,149]. In systemic circulation, the function of myostatin is inhibited by binding to follistatin [150].

In animal models with uremia, myostatin role inhibition has been attempted to attenuate protein catabolism. For instance, anti-myostatin peptibody that was generated as a chimeric peptide-Fc fusion protein was used to treat CKD mice for four weeks resulting in improvement of body weight and less muscle mass loss. At the same time, muscle protein degradation is reduced while, intriguingly, protein synthesis and satellite cell function are enhanced [92]. The inhibition of myostatin is also helpful in preventing the development of fibrosis in skeletal muscle. Administration of myostatin neutralizing peptibody to CKD rodents reduced the proliferation of fibroblast progenitors and muscle fibrosis [95].

In humans, the frail elderly showed elevated blood levels of GDF11 that are highly homologous to GDF8, myostatin [151]. Thus, the myostatin receptor inhibitory signals are hypothesized to exert anabolic effects to enhance skeletal muscle strength in sarcopenia [152,153,154]. Although not applied for uremic sarcopenia, a phase 1 study of anti-myostatin monoclonal antibody LY2495655 in patients with advanced cancer was presented. The treatment improved handgrip strength and physical function scoring. In a clinical study evaluating the effects of the compound in elderly frail people [152], a lean body mass at 24 weeks was slightly increased, with several measures of improved muscle function. In another trial to assess the safety and efficacy of the agent for 24 weeks after elective total hip arthroplasty [153], expected alteration of lean body mass and fat mass was not confirmed.

### 5.4. Activation of Ghrelin Signaling

Ghrelin is mainly produced in gastric endocrine cells and has essential effects on energy metabolism regulation, such as increased appetite, weight gain, and gastrointestinal digestive function [155]. The peptide hormone is modified by fatty acid and promotes muscle mitochondrial oxidation. Intraperitoneal administration of acylated ghrelin (0.1 μmol/kg) three times per week to subtotal nephrectomized mice improved a decline in treadmill exercise endurance, associated with an increase in both the muscle mass and mitochondrial amounts [114]. The treatment decreased the methylation ratio of the promoter region of PGC-1α in the skeletal muscle, leading to increased mitochondrial content. Additionally, treatment with unacylated ghrelin enhanced mitophagy and normalized oxidative stress in the GC muscle in 5/6 nephrectomized rats. The treatment alsoattenuated inflammation, insulin resistance, and muscle loss [99].

Anamorelin is developed as a small molecule to enhance protein synthesis by binding the ghrelin/growth hormone secretagogue receptor (GHSR), aiming at the treatment of malignant cachexia [156,157,158]. The drug effect would be divergent in the case that clinical trials for uremic failure is conducted.

### 5.5. Incretin Modulation

Both glucagon-like peptide-1 (GLP-1) receptor agonist and dipeptidyl peptidase 4 (DPP-4) inhibitors enhance incretin levels, which inhibit glucagon release, which in turn, increases insulin secretion, as a class of oral hypoglycemics used to treat diabetes mellitus type 2. Intraperitoneal administration of exendin-4 (100 ng/day), a GLP-1 receptor agonist, for 12 days [89] or oral administration of teneligliptin (60 mg/kg), a DPP-4 inhibitor, for 20 weeks [97], ameliorated body weight loss and muscle atrophy, and improved grip strength using mice with subtotal nephrectomy.

### 5.6. Carbon Sorbent

In mice with subtotal nephrectomy, the IS level in the circulating blood is elevated, along with a shortening of the running distance to exhaustion determined by treadmill tests, but oral carbon sorbent administration prevented this increase [159]. After four weeks of oral treatment with 8% (*w*/*w*) AST-120, muscle atrophy was ameliorated in subtotal nephrectomized mice. Since it theoretically binds and partially prevents uremic toxins from being absorbed by the gastrointestinal tract, the sorbent exerts a beneficial effect by reducing systemic accumulation of uremic toxins. Given that the excessive accumulation of uremic toxins confer multiple organ dysfunction in uremia, the sorbent may be useful for treating uremic sarcopenia [23,97]. Despite animal studies supporting a renoprotective effect of the drug [160,161], human clinical trials, including recent ones, EPPIC-1 and EPPIC-2, have not shown a conclusive beneficial effect [162,163,164] and the drug was less widely adopted by nephrologists compared to other drugs with an established effect on CKD symptoms, such as anemia or electrolyte disorders [165]. However, suboptimal eradication of the toxin can be a reason for failure when encountering negative results.

### 5.7. Erythropoiesis Stimulating Agent

Renal anemia is a typical complication of CKD and has been treated with recombinant human erythropoietin (EPO) preparations [166]. EPO and the receptor have been well documented to transmit signals towards protein kinases, transcription factors, and anti-apoptotic proteins [167,168]. Indeed, the presence and bioactivity of the receptor in non-hematopoietic tissues implies that EPO exerts a pleiotropic effect in various tissues and organs [169,170]. Intriguingly, endurance time as assessed with a rotarod test in bilateral IRI mice was improved with EPO (1000 U/kg) intraperitoneally injected 30 min prior to surgery [110]. Additionally, prolyl hydroxylase domain (HIF-PH) inhibitors, which are expected to be erythropoiesis-stimulating agents, are a new class of compounds that focus on enhancing endogenous EPO production [171]. Interestingly, a decrease in skeletal muscle mass, mitochondrial amount, and exercise capacity in subtotal nephrectomized mice were restored by a 12 week treatment with an orally active HIF-PH inhibitor, MK-8617 (1.5 to 12.5 mg/kg). Increased capillary density in skeletal muscle is ancillary to muscle function improvement, which may be caused by the upregulation of HIF-downstream VEGF expression [102]. Our laboratory also focused on the pharmacological effect of HIF-PH inhibitor on skeletal myocytes and tissue and also clarified that the pharmacological treatment enhanced glycolysis and suppressed the tricarboxylic acid (TCA) cycle, thus mimicking the Walberg effect in tumors (Takemura, Nishi, unpublished).

### 5.8. Lipotoxicity Eradication

Lipotoxicity is defined as ectopic fat distribution in the peripheral organs with subsequent injurious effects in end organs, including the kidney, [172,173,174,175] and skeletal muscle [176,177,178]. In terms of cellular energy production, long-chain fatty acyl-CoA is esterified with carnitine in the cytoplasm, and then, the carnitine shuttle serves to carry it from the cytosol to the mitochondria, and thus, the carnitine nutritional supplement is expected to enhance or normalize long-chain fatty acid metabolism. The administration of L-carnitine (560 mg/kg) for 24 weeks significantly suppressed this impaired exercise capacity. Subtotal nephrectomy in mice induces lowered expression of PGC-1α, autophagy induction, and even muscle fiber switch from type I to type II in the GC muscle, all of which were restored with L-carnitine treatment [97].

### 5.9. Small RNA and Lnc RNA

Given that RNA has been approved as the newest therapeutic modality in humans [179], these approaches can provide options for treating uremic sarcopenia in the future. By utilizing STZ-induced kidney injury and uremic sarcopenia in mice, the intramuscular injection of adeno-associated virus (AAV)-miR-23a/27a to TA reduced the abundance of atrgoin-1 and MuRF-1 in skeletal muscles. In addition, this viral vector injection attenuated the diabetes-induced reduction in muscle CSA and muscle function along with attenuation of renal function and fibrosis [108]. Long noncoding RNAs (lncRNAs) are defined as >200 nucleotide RNAs lacking the protein-coding potential. Besides, lncRNA arrays identified Atrolnc-1 as one gene that is robustly elevated in atrophying muscles from mice with cachexia. They found that depressed insulin signaling stimulates the transcription factor C/EBP-α in binding to the promoter of Atrolnc-1 and promotes the expression of Atrolnc-1. It interacts with the A20 binding inhibitor of NF-κB-1 (ABIN-1), resulting in enhanced NF-κB activity plus MuRF-1 transcription, leading to muscle atrophy. Surprisingly, AAV mediated silencing of Atrolnc-1 in TA muscle in mice with subtotal nephrectomy and restored the ratio of muscle weight to tibia bone length and CSA of the myofibers [100].

## 6. Conclusions

Clinicians should be aware that sarcopenia occurs not only in the elderly but also secondarily, due to chronic illnesses such as cancer, heart failure, and CKD, and negatively affects daily physical activity. When found in coexistence with CKD, the presence of sarcopenia can be a life prognostic factor. The measures that can be taken to improve this condition are dietary therapy and safe exercise. However, it has yet to be determined how much and what kinds of exercise will benefit individual patients at various stages of disease and fitness. There is an urgent need for an appropriate experimental model that can verify the pathology and determine treatment of uremic sarcopenia. Further research in the field will yield a new clinical and biological evidence to comprehensively understand the mechanisms and mediators of interaction between the skeletal muscle and the kidney, which may alter recommendations for diet and exercise strategies against CKD.

## Figures and Tables

**Table 1:** Exercise therapy approach to patients with various stages of CKD.

	Skeletal Muscle Strength	VO_2_ Peak/VO_2_ Max	6-Min Walk	Physical Activity (QOL)	GFR Reduction	Hospitalization	Mortality
Pre-dialysis CKD	+ [44,45,46,47]	+ * [48,49,50,51]	+ * [52,53]	+ * [52,54]	+ * [55]	→ * [56]	→ * [56]
Hemodialysis	+ [47,57,58]	+ [59,60,61,62,63,64,65,66]	+ [67]	+ [68,69,70]	NA	Unknown	Unknown
Kidney transplant recipients	→ [71]	+ [72]	Unknown	+ [72,73]	→ [74,75]	Unknown	Unknown

* analyzed in mainly pre-dialysis obese CKD patients with DKD. CKD: chronic kidney disease, QOL: quality of life, DKD: diabetic kidney disease: NA: not applicable

**Table 2:** Experimental animal models: implication in uremic sarcopenia.

	Skeletal Muscle Manifestation	Literature
Subtotal (5/6) nephrectomy	Muscle atrophy ↓Treadmill running endurance ↓Grip strength, hanging time on the grid ↓Overnight ambulation ↓Swimming velocity ↑Expression of myostatin, atrogin-1, MuRF-1, IL-6, Binp3, Beclin-1, LC3II ↓Expression of PGC1α, andAMPK	[89,90,91,92,93,94,95,96,97,98,99,100,101,102]
Adenine diet administration	Muscle atrophy ↓Concentration of ATP and intracellular phosphate	[103,104]
Indoxyl sulfate administration	Muscle atrophy ↑Expression of myostatin, atrogin-1, IL-6, TNF-α	[105]
Cy/+ rat (Han:sPRD rat with polycystic kidney disease)	↓Maximum torque in ankle dorsiflexion ↑Relaxation time in hind limb dorsiflexion and plantarflexion ↑Expression of atrogin-1, MuRF-1, oxidative stress markers	[106,107]
Diabetic kidney disease	Muscle atrophy ↓Muscle regeneration ↑Expression of myostatin, atrogin-1, MuRF-1	[108,109]
Kidney ischemia-reperfusion injury	↓Motor coordination and balance	[110]

↑: increased or enhanced, ↓: decreased or deteriorated.

## References

[1] Cruz-Jentoft A.J., Baeyens J.P., Bauer J.M., Boirie Y., Cederholm T., Landi F., Martin F.C., Michel J.P., Rolland Y., Schneider S.M. (2010). Sarcopenia: European consensus on definition and diagnosis: Report of the European Working Group on Sarcopenia in Older People. Age Ageing.

[2] Epidemiologic and methodologic problems in determining nutritional status of older persons (1989). Proceedings of a conference. Albuquerque, New Mexico, October 19–21, 1988. Am. J. Clin. Nutr..

[3] Cruz-Jentoft A.J., Bahat G., Bauer J., Boirie Y., Bruyère O., Cederholm T., Cooper C., Landi F., Rolland Y., Sayer A.A. (2019). Sarcopenia: Revised European consensus on definition and diagnosis. Age Ageing.

[4] Chen L.-K., Liu L.-K., Woo J., Assantachai P., Auyeung T.-W., Bahyah K.S., Chou M.-Y., Chen L.-Y., Hsu P.-S., Krairit O. (2014). Sarcopenia in Asia: Consensus report of the Asian Working Group for Sarcopenia. J. Am. Med. Dir. Assoc..

[5] Moorthi R.N., Avin K.G. (2017). Clinical relevance of sarcopenia in chronic kidney disease. Curr. Opin. Nephrol. Hypertens..

[6] Foley R.N., Wang C., Ishani A., Collins A.J., Murray A.M. (2007). Kidney function and sarcopenia in the United States general population: NHANES III. Am. J. Nephrol..

[7] Roshanravan B., Patel K.V., Robinson-Cohen C., de Boer I.H., O’Hare A.M., Ferrucci L., Himmelfarb J., Kestenbaum B. (2015). Creatinine clearance, walking speed, and muscle atrophy: A cohort study. Am. J. Kidney Dis..

[8] Ikizler T.A., Greene J.H., Wingard R.L., Parker R.A., Hakim R.M. (1995). Spontaneous dietary protein intake during progression of chronic renal failure. J. Am. Soc. Nephrol..

[9] Moon S.J., Kim T.H., Yoon S.Y., Chung J.H., Hwang H.J. (2015). Relationship between Stage of Chronic Kidney Disease and Sarcopenia in Korean Aged 40 Years and Older Using the Korea National Health and Nutrition Examination Surveys (KNHANES IV-2, 3, and V-1, 2), 2008–2011. PLoS ONE.

[10] Sinkeler S.J., Kwakernaak A.J., Bakker S.J., Shahinfar S., Esmatjes E., de Zeeuw D., Navis G., Lambers Heerspink H.J. (2013). Creatinine excretion rate and mortality in type 2 diabetes and nephropathy. Diabetes Care..

[11] Pereira R.A., Cordeiro A.C., Avesani C.M., Carrero J.J., Lindholm B., Amparo F.C., Amodeo C., Cuppari L., Kamimura M.A. (2015). Sarcopenia in chronic kidney disease on conservative therapy: Prevalence and association with mortality. Nephrol. Dial. Trans..

[12] Roshanravan B., Robinson-Cohen C., Patel K.V., Ayers E., Littman A.J., de Boer I.H., Ikizler T.A., Himmelfarb J., Katzel L.I., Kestenbaum B. (2013). Association between physical performance and all-cause mortality in CKD. J. Am. Soc. Nephrol..

[13] Sharma D., Hawkins M., Abramowitz M.K. (2014). Association of sarcopenia with eGFR and misclassification of obesity in adults with CKD in the United States. Clin. J. Am. Soc Nephrol..

[14] Kim J.K., Choi S.R., Choi M.J., Kim S.G., Lee Y.K., Noh J.W., Kim H.J., Song Y.R. (2014). Prevalence of and factors associated with sarcopenia in elderly patients with end-stage renal disease. Clin. Nutr..

[15] Morishita Y., Kubo K., Miki A., Ishibashi K., Kusano E., Nagata D. (2014). Positive association of vigorous and moderate physical activity volumes with skeletal muscle mass but not bone density or metabolism markers in hemodialysis patients. Int. Urol. Nephrol..

[16] Hanatani S., Izumiya Y., Onoue Y., Tanaka T., Yamamoto M., Ishida T., Yamamura S., Kimura Y., Araki S., Arima Y. (2018). Non-invasive testing for sarcopenia predicts future cardiovascular events in patients with chronic kidney disease. Int. J. Cardiol..

[17] Huang C.X., Tighiouart H., Beddhu S., Cheung A.K., Dwyer J.T., Eknoyan G., Beck G.J., Levey A.S., Sarnak M.J. (2010). Both low muscle mass and low fat are associated with higher all-cause mortality in hemodialysis patients. Kidney Int..

[18] Stenvinkel P., Alvestrand A. (2002). Inflammation in end-stage renal disease: Sources, consequences, and therapy. Semin. Dial..

[19] Delano M.J., Moldawer L.L. (2006). The origins of cachexia in acute and chronic inflammatory diseases. Nutr. Clin. Pract..

[20] Ishikawa S., Naito S., Iimori S., Takahashi D., Zeniya M., Sato H., Nomura N., Sohara E., Okado T., Uchida S. (2018). Loop diuretics are associated with greater risk of sarcopenia in patients with non-dialysis-dependent chronic kidney disease. PLoS ONE.

[21] Honda H., Qureshi A.R., Axelsson J., Heimburger O., Suliman M.E., Barany P., Stenvinkel P., Lindholm B. (2007). Obese sarcopenia in patients with end-stage renal disease is associated with inflammation and increased mortality. Am. J. Clin. Nutr..

[22] Yuan J., Watanabe M., Suliman M., Qureshi A.R., Axelsson J., Barany P., Heimburger O., Stenvinkel P., Lindholm B. (2015). Serum hepatocyte growth factor is associated with truncal fat mass and increased mortality in chronic kidney disease stage 5 patients with protein-energy wasting. Nephrol. Dial. Trans..

[23] Sato E., Saigusa D., Mishima E., Uchida T., Miura D., Morikawa-Ichinose T., Kisu K., Sekimoto A., Saito R., Oe Y. (2017). Impact of the Oral Adsorbent AST-120 on Organ-Specific Accumulation of Uremic Toxins: LC-MS/MS and MS Imaging Techniques. Toxins.

[24] Androga L., Sharma D., Amodu A., Abramowitz M.K. (2017). Sarcopenia, obesity, and mortality in US adults with and without chronic kidney disease. Kidney Int. Rep..

[25] Cohen I.M., Griffiths J., Stone R.A., Leech T. (1980). The creatine kinase profile of a maintenance hemodialysis population: A possible marker of uremic myopathy. Clin. Nephrol..

[26] Campistol J.M. (2002). Uremic myopathy. Kidney Int..

[27] Pedrini M.T., Levey A.S., Lau J., Chalmers T.C., Wang P.H. (1996). The effect of dietary protein restriction on the progression of diabetic and nondiabetic renal diseases: A meta-analysis. Ann. Intern. Med..

[28] Fouque D., Laville M. (2006). Low protein diets for chronic kidney disease in non diabetic adults. Cochrane Database Syst. Rev..

[29] Klahr S., Levey A.S., Beck G.J., Caggiula A.W., Hunsicker L., Kusek J.W., Striker G. (1994). The effects of dietary protein restriction and blood-pressure control on the progression of chronic renal disease. Modification of Diet in Renal Disease Study Group. N. Engl. J. Med..

[30] Levey A.S., Greene T., Beck G.J., Caggiula A.W., Kusek J.W., Hunsicker L.G., Klahr S. (1999). Dietary protein restriction and the progression of chronic renal disease: What have all of the results of the MDRD study shown? Modification of Diet in Renal Disease Study group. J. Am. Soc. Nephrol..

[31] Kalantar-Zadeh K., Fouque D. (2017). Nutritional Management of Chronic Kidney Disease. N. Engl. J. Med..

[32] Mircescu G., Garneata L., Stancu S.H., Capusa C. (2007). Effects of a supplemented hypoproteic diet in chronic kidney disease. J. Ren. Nutr..

[33] Brunori G., Viola B.F., Parrinello G., De Biase V., Como G., Franco V., Garibotto G., Zubani R., Cancarini G.C. (2007). Efficacy and safety of a very-low-protein diet when postponing dialysis in the elderly: A prospective randomized multicenter controlled study. Am. J. Kidney Dis..

[34] National Kidney F. (2002). K/DOQI clinical practice guidelines for chronic kidney disease: Evaluation, classification, and stratification. Am. J. Kidney Dis..

[35] Bauer J., Biolo G., Cederholm T., Cesari M., Cruz-Jentoft A.J., Morley J.E., Phillips S., Sieber C., Stehle P., Teta D. (2013). Evidence-based recommendations for optimal dietary protein intake in older people: A position paper from the PROT-AGE Study Group. J. Am. Med. Dir. Assoc..

[36] Hays R.D., Kallich J.D., Mapes D.L., Coons S.J., Carter W.B. (1994). Development of the kidney disease quality of life (KDQOL) instrument. Qual. Life Res..

[37] Deutz N.E., Bauer J.M., Barazzoni R., Biolo G., Boirie Y., Bosy-Westphal A., Cederholm T., Cruz-Jentoft A., Krznaric Z., Nair K.S. (2014). Protein intake and exercise for optimal muscle function with aging: Recommendations from the ESPEN Expert Group. Clin. Nutr..

[38] Xu Z.R., Tan Z.J., Zhang Q., Gui Q.F., Yang Y.M. (2014). Clinical effectiveness of protein and amino acid supplementation on building muscle mass in elderly people: A meta-analysis. PLoS ONE.

[39] Xu Z.R., Tan Z.J., Zhang Q., Gui Q.F., Yang Y.M. (2015). The effectiveness of leucine on muscle protein synthesis, lean body mass and leg lean mass accretion in older people: A systematic review and meta-analysis. Br. J. Nutr..

[40] Komar B., Schwingshackl L., Hoffmann G. (2015). Effects of leucine-rich protein supplements on anthropometric parameter and muscle strength in the elderly: A systematic review and meta-analysis. J. Nutr. Health Aging.

[41] Wu H., Xia Y., Jiang J., Du H., Guo X., Liu X., Li C., Huang G., Niu K. (2015). Effect of beta-hydroxy-beta-methylbutyrate supplementation on muscle loss in older adults: A systematic review and meta-analysis. Arch. Gerontol. Geriatr..

[42] Yoshimura Y., Wakabayashi H., Yamada M., Kim H., Harada A., Arai H. (2017). Interventions for Treating Sarcopenia: A Systematic Review and Meta-Analysis of Randomized Controlled Studies. J. Am. Med. Dir. Assoc..

[43] Tieland M., Franssen R., Dullemeijer C., van Dronkelaar C., Kyung Kim H., Ispoglou T., Zhu K., Prince R.L., van Loon L.J.C., de Groot L. (2017). The Impact of Dietary Protein or Amino Acid Supplementation on Muscle Mass and Strength in Elderly People: Individual Participant Data and Meta-Analysis of RCT’s. J. Nutr. Health Aging.

[44] Hiraki K., Shibagaki Y., Izawa K.P., Hotta C., Wakamiya A., Sakurada T., Yasuda T., Kimura K. (2017). Effects of home-based exercise on pre-dialysis chronic kidney disease patients: A randomized pilot and feasibility trial. BMC Nephrol..

[45] Hellberg M., Hoglund P., Svensson P., Clyne N. (2018). Comparing effects of 4 months of two self-administered exercise training programs on physical performance in patients with chronic kidney disease: RENEXC—A randomized controlled trial. PLoS ONE.

[46] Watson E.L., Greening N.J., Viana J.L., Aulakh J., Bodicoat D.H., Barratt J., Feehally J., Smith A.C. (2015). Progressive Resistance Exercise Training in CKD: A Feasibility Study. Am. J. Kidney Dis..

[47] Olvera-Soto M.G., Valdez-Ortiz R., Lopez Alvarenga J.C., Espinosa-Cuevas Mde L. (2016). Effect of Resistance Exercises on the Indicators of Muscle Reserves and Handgrip Strength in Adult Patients on Hemodialysis. J. Ren. Nutr..

[48] Mustata S., Groeneveld S., Davidson W., Ford G., Kiland K., Manns B. (2011). Effects of exercise training on physical impairment, arterial stiffness and health-related quality of life in patients with chronic kidney disease: A pilot study. Int. Urol. Nephrol..

[49] Howden E.J., Leano R., Petchey W., Coombes J.S., Isbel N.M., Marwick T.H. (2013). Effects of exercise and lifestyle intervention on cardiovascular function in CKD. Clin. J. Am. Soc. Nephrol..

[50] Van Craenenbroeck A.H., Van Craenenbroeck E.M., Van Ackeren K., Vrints C.J., Conraads V.M., Verpooten G.A., Kouidi E., Couttenye M.M. (2015). Effect of Moderate Aerobic Exercise Training on Endothelial Function and Arterial Stiffness in CKD Stages 3–4: A Randomized Controlled Trial. Am. J. Kidney Dis..

[51] Greenwood S.A., Koufaki P., Mercer T.H., Maclaughlin H.L., Rush R., Lindup H., Connor E., Jones C., Hendry B.M., Macdougall I.C. (2015). Effect of exercise training on estimated GFR, vascular health, and cardiorespiratory fitness in patients with CKD: A pilot randomized controlled trial. Am. J. Kidney Dis..

[52] Rossi A.P., Burris D.D., Lucas F.L., Crocker G.A., Wasserman J.C. (2014). Effects of a renal rehabilitation exercise program in patients with CKD: A randomized, controlled trial. Clin. J. Am. Soc. Nephrol..

[53] Howden E.J., Coombes J.S., Strand H., Douglas B., Campbell K.L., Isbel N.M. (2015). Exercise training in CKD: Efficacy, adherence, and safety. Am. J. Kidney Dis..

[54] Headley S., Germain M., Wood R., Joubert J., Milch C., Evans E., Poindexter A., Cornelius A., Brewer B., Pescatello L.S. (2014). Short-term aerobic exercise and vascular function in CKD stage 3: A randomized controlled trial. Am. J. Kidney Dis..

[55] Look ARG (2014). Effect of a long-term behavioural weight loss intervention on nephropathy in overweight or obese adults with type 2 diabetes: A secondary analysis of the Look AHEAD randomised clinical trial. Lancet Diabetes Endocrinol..

[56] Gong Q., Gregg E.W., Wang J., An Y., Zhang P., Yang W., Li H., Li H., Jiang Y., Shuai Y. (2011). Long-term effects of a randomised trial of a 6-year lifestyle intervention in impaired glucose tolerance on diabetes-related microvascular complications: The China Da Qing Diabetes Prevention Outcome Study. Diabetologia.

[57] Simo V.E., Jiménez A.J., Guzmán F.M., Oliveira J.C., Nicolas M.F., Potau M.P., Sole A.S., Gallego V.D., Gonzalez I.T., de Arellano M.R. (2015). Benefits of a low intensity exercise programme during haemodialysis sessions in elderly patients. Nefrologia.

[58] Dong Z.J., Zhang H.L., Yin L.X. (2019). Effects of intradialytic resistance exercise on systemic inflammation in maintenance hemodialysis patients with sarcopenia: A randomized controlled trial. Int. Urol. Nephrol..

[59] Koufaki P., Mercer T.H., Naish P.F. (2002). Effects of exercise training on aerobic and functional capacity of end-stage renal disease patients. Clin. Physiol. Funct. Imaging.

[60] Petraki M., Kouidi E., Grekas D., Deligiannis A. (2008). Effects of exercise training during hemodialysis on cardiac baroreflex sensitivity. Clin. Nephrol..

[61] Ouzouni S., Kouidi E., Sioulis A., Grekas D., Deligiannis A. (2009). Effects of intradialytic exercise training on health-related quality of life indices in haemodialysis patients. Clin. Rehabil..

[62] Kouidi E.J., Grekas D.M., Deligiannis A.P. (2009). Effects of exercise training on noninvasive cardiac measures in patients undergoing long-term hemodialysis: A randomized controlled trial. Am. J. Kidney Dis..

[63] Kouidi E., Karagiannis V., Grekas D., Iakovides A., Kaprinis G., Tourkantonis A., Deligiannis A. (2010). Depression, heart rate variability, and exercise training in dialysis patients. Eur. J. Cardiovasc. Prev. Rehabil..

[64] Reboredo M.M., Neder J.A., Pinheiro B.V., Henrique D.M., Faria R.S., Paula R.B. (2011). Constant work-rate test to assess the effects of intradialytic aerobic training in mildly impaired patients with end-stage renal disease: A randomized controlled trial. Arch. Phys. Med. Rehabil..

[65] Kouidi E., Iacovides A., Iordanidis P., Vassiliou S., Deligiannis A., Ierodiakonou C., Tourkantonis A. (1997). Exercise renal rehabilitation program: Psychosocial effects. Nephron.

[66] Deligiannis A., Kouidi E., Tourkantonis A. (1999). Effects of physical training on heart rate variability in patients on hemodialysis. Am. J. Cardiol..

[67] Pellizzaro C.O., Thome F.S., Veronese F.V. (2013). Effect of peripheral and respiratory muscle training on the functional capacity of hemodialysis patients. Ren. Fail..

[68] Song W.J., Sohng K.Y. (2012). Effects of progressive resistance training on body composition, physical fitness and quality of life of patients on hemodialysis. J. Korean Acad. Nurs..

[69] Dobsak P., Homolka P., Svojanovsky J., Reichertova A., Soucek M., Novakova M., Dusek L., Vasku J., Eicher J.-C., Siegelova J. (2012). Intra-dialytic electrostimulation of leg extensors may improve exercise tolerance and quality of life in hemodialyzed patients. Artif. Organs..

[70] Hristea D., Deschamps T., Paris A., Lefrançois G., Collet V., Savoiu C., Ozenne S., Coupel S., Testa A., Magnard J. (2016). Combining intra-dialytic exercise and nutritional supplementation in malnourished older haemodialysis patients: Towards better quality of life and autonomy. Nephrology.

[71] Henggeler C.K., Plank L.D., Ryan K.J., Gilchrist E.L., Casas J.M., Lloyd L.E., Mash L.E., McLellan S.L., Robb J.M., Collins M.G. (2018). A Randomized Controlled Trial of an Intensive Nutrition Intervention Versus Standard Nutrition Care to Avoid Excess Weight Gain After Kidney Transplantation: The INTENT Trial. J. Ren. Nutr..

[72] Kouidi E., Vergoulas G., Anifanti M., Deligiannis A. (2013). A randomized controlled trial of exercise training on cardiovascular and autonomic function among renal transplant recipients. Nephrol. Dial. Trans..

[73] Riess K.J., Haykowsky M., Lawrance R., Tomczak C.R., Welsh R., Lewanczuk R., Tymchak W., Haennel R.G., Gourishankar S. (2014). Exercise training improves aerobic capacity, muscle strength, and quality of life in renal transplant recipients. Appl. Physiol. Nutr. Metab..

[74] Tzvetanov I., West-Thielke P., D’Amico G., Johnsen M., Ladik A., Hachaj G., Grazman M., Heller R.U., Fernhall B., Daviglus M.L. (2014). A novel and personalized rehabilitation program for obese kidney transplant recipients. Trans. Proc..

[75] Greenwood S.A., Koufaki P., Mercer T.H., Rush R., O’Connor E., Tuffnell R., Lindup H., Haggis L., Dew T., Abdulnassir L. (2015). Aerobic or Resistance Training and Pulse Wave Velocity in Kidney Transplant Recipients: A 12-Week Pilot Randomized Controlled Trial (the Exercise in Renal Transplant [ExeRT] Trial). Am. J. Kidney Dis..

[76] Cheema B., Abas H., Smith B., O’Sullivan A., Chan M., Patwardhan A., Kelly J., Gillin A., Pang G., Lloyd B. (2007). Progressive exercise for anabolism in kidney disease (PEAK): A randomized, controlled trial of resistance training during hemodialysis. J. Am. Soc. Nephrol..

[77] Cheema B., Abas H., Smith B., O’Sullivan A., Chan M., Patwardhan A., Kelly J., Gillin A., Pang G., Lloyd B. (2007). Randomized controlled trial of intradialytic resistance training to target muscle wasting in ESRD: The Progressive Exercise for Anabolism in Kidney Disease (PEAK) study. Am. J. Kidney Dis..

[78] Giannaki C.D., Sakkas G.K., Karatzaferi C., Hadjigeorgiou G.M., Lavdas E., Kyriakides T., Koutedakis Y., Stefanidis I. (2013). Effect of exercise training and dopamine agonists in patients with uremic restless legs syndrome: A six-month randomized, partially double-blind, placebo-controlled comparative study. BMC Nephrol..

[79] Koh K.P., Fassett R.G., Sharman J.E., Coombes J.S., Williams A.D. (2010). Effect of intradialytic versus home-based aerobic exercise training on physical function and vascular parameters in hemodialysis patients: A randomized pilot study. Am. J. Kidney Dis..

[80] Wu Y., He Q., Yin X., He Q., Cao S., Ying G. (2014). Effect of individualized exercise during maintenance haemodialysis on exercise capacity and health-related quality of life in patients with uraemia. J. Int. Med. Res..

[81] Sheng K., Zhang P., Chen L., Cheng J., Wu C., Chen J. (2014). Intradialytic exercise in hemodialysis patients: A systematic review and meta-analysis. Am. J. Nephrol..

[82] Manfredini F., Lamberti N., Malagoni A.M., Felisatti M., Zuccala A., Torino C., Tripepi G., Catizone L., Mallamaci F., Zoccali C. (2015). The role of deconditioning in the end-stage renal disease myopathy: Physical exercise improves altered resting muscle oxygen consumption. Am. J. Nephrol..

[83] Ono Y., Sensui H., Sakamoto Y., Nagatomi R. (2006). Knockdown of hypoxia-inducible factor-1alpha by siRNA inhibits C2C12 myoblast differentiation. J. Cell Biochem..

[84] Manabe Y., Miyatake S., Takagi M., Nakamura M., Okeda A., Nakano T., Hirshman M.F., Goodyear L.J., Fujii N.L. (2012). Characterization of an acute muscle contraction model using cultured C2C12 myotubes. PLoS ONE.

[85] Abdelmoez A.M., Sardon Puig L., Smith J.A.B., Gabriel B.M., Savikj M., Dollet L., Chibalin A.V., Krook A., Zierath J.R. (2020). Comparative profiling of skeletal muscle models reveals heterogeneity of transcriptome and metabolism. Am. J. Physiol. Cell. Physiol..

[86] Ono Y., Boldrin L., Knopp P., Morgan J.E., Zammit P.S. (2010). Muscle satellite cells are a functionally heterogeneous population in both somite-derived and branchiomeric muscles. Dev. Biol..

[87] Manabe Y., Gollisch K.S., Holton L., Kim Y.B., Brandauer J., Fujii N.L., Hirshman M.F., Goodyear L.J. (2013). Exercise training-induced adaptations associated with increases in skeletal muscle glycogen content. FEBS J..

[88] Ishida J., Saitoh M., Doehner W., von Haehling S., Anker M., Anker S.D., Springer J., Döhner W. (2017). Animal models of cachexia and sarcopenia in chronic illness: Cardiac function, body composition changes and therapeutic results. Int. J. Cardiol..

[89] Hong Y., Lee J.H., Jeong K.W., Choi C.S., Jun H.S. (2019). Amelioration of muscle wasting by glucagon-like peptide-1 receptor agonist in muscle atrophy. J. Cachexia Sarcopenia Muscle.

[90] Al Banchaabouchi M., D’Hooge R., Marescau B., De Deyn P.P. (1999). Behavioural deficits during the acute phase of mild renal failure in mice. Metab. Brain Dis..

[91] Wang X.H., Du J., Klein J.D., Bailey J.L., Mitch W.E. (2009). Exercise ameliorates chronic kidney disease-induced defects in muscle protein metabolism and progenitor cell function. Kidney Int..

[92] Zhang L., Rajan V., Lin E., Hu Z., Han H.Q., Zhou X., Song Y., Min H., Wang X., Du J. (2011). Pharmacological inhibition of myostatin suppresses systemic inflammation and muscle atrophy in mice with chronic kidney disease. FASEB J..

[93] Tamaki M., Miyashita K., Wakino S., Mitsuishi M., Hayashi K., Itoh H. (2014). Chronic kidney disease reduces muscle mitochondria and exercise endurance and its exacerbation by dietary protein through inactivation of pyruvate dehydrogenase. Kidney Int..

[94] Hu L., Klein J.D., Hassounah F., Cai H., Zhang C., Xu P., Wang X.H. (2015). Low-frequency electrical stimulation attenuates muscle atrophy in CKD--a potential treatment strategy. J. Am. Soc. Nephrol..

[95] Dong J., Dong Y., Chen Z., Mitch W.E., Zhang L. (2017). The pathway to muscle fibrosis depends on myostatin stimulating the differentiation of fibro/adipogenic progenitor cells in chronic kidney disease. Kidney Int..

[96] Yu R., Chen J.A., Xu J., Cao J., Wang Y., Thomas S.S., Hu Z.Y. (2017). Suppression of muscle wasting by the plant-derived compound ursolic acid in a model of chronic kidney disease. J. Cachexia Sarcopenia Muscle.

[97] Enoki Y., Watanabe H., Arake R., Fujimura R., Ishiodori K., Imafuku T., Nishida K., Sugimoto R., Nagao S., Miyamura S. (2017). Potential therapeutic interventions for chronic kidney disease-associated sarcopenia via indoxyl sulfate-induced mitochondrial dysfunction. J. Cachexia Sarcopenia Muscle.

[98] Su Z., Klein J.D., Du J., Franch H.A., Zhang L., Hassounah F., Hudson M.B., Wang X.H. (2017). Chronic kidney disease induces autophagy leading to dysfunction of mitochondria in skeletal Muscle. Am. J. Physiol. Renal. Physiol..

[99] Gortan Cappellari G., Semolic A., Ruozi G., Vinci P., Guarnieri G., Bortolotti F., Barbetta D., Zanetti M., Giacca M., Barazzoni R. (2017). Unacylated ghrelin normalizes skeletal muscle oxidative stress and prevents muscle catabolism by enhancing tissue mitophagy in experimental chronic kidney disease. FASEB J..

[100] Sun L., Si M., Liu X., Choi J.M., Wang Y., Thomas S.S., Peng H., Hu Z. (2018). Long-noncoding RNA Atrolnc-1 promotes muscle wasting in mice with chronic kidney disease. J. Cachexia Sarcopenia Muscle.

[101] Kikuchi H., Sasaki E., Nomura N., Mori T., Minamishima Y.A., Yoshizaki Y., Takahashi N., Furusho T., Arai Y., Mandai S. (2019). Failure to sense energy depletion may be a novel therapeutic target in chronic kidney disease. Kidney Int..

[102] Qian F.Y., Li Z.L., Guo Y.D., Gao H.C., Gu L.H., Le K., Xie C.M., Wang B., Zhang Z.J. (2019). Hypoxia-inducible factor-prolyl hydroxylase inhibitor ameliorates myopathy in a mouse model of chronic kidney disease. Am. J. Physiol. Renal. Physiol..

[103] Sato E., Mori T., Mishima E., Suzuki A., Sugawara S., Kurasawa N., Saigusa D., Miura D., Morikawa-Ichinose T., Saito R. (2016). Metabolic alterations by indoxyl sulfate in skeletal muscle induce uremic sarcopenia in chronic kidney disease. Sci. Rep..

[104] Andres-Hernando A., Lanaspa M.A., Kuwabara M., Orlicky D.J., Cicerchi C., Bales E., Garcia G.E., Roncal-Jimenez C.A., Sato Y., Johnson R.J. (2019). Obesity causes renal mitochondrial dysfunction and energy imbalance and accelerates chronic kidney disease in mice. Am. J. Physiol. Renal. Physiol..

[105] Enoki Y., Watanabe H., Arake A., Sugimoto R., Imafuku T., Tominaga Y., Ishima Y., Kotani S., Nakajima M., Tanaka M. (2016). Indoxyl sulfate potentiates skeletal muscle atrophy by inducing the oxidative stress-mediated expression of myostatin and atrogin-1. Sci. Rep..

[106] Organ J.M., Srisuwananukorn A., Price P., Joll J.E., Biro K.C., Rupert J.E., Chen N.X., Avin K.G., Moe S.M., Allen M.R. (2016). Reduced skeletal muscle function is associated with decreased fiber cross-sectional area in the Cy/+ rat model of progressive kidney disease. Nephrol. Dial. Trans..

[107] Avin K.G., Chen N.X., Organ J.M., Zarse C., O’Neill K., Conway R.G., Konrad R.J., Bacallao R.L., Allen M.R., Moe S.M. (2016). Skeletal Muscle Regeneration and Oxidative Stress Are Altered in Chronic Kidney Disease. PLoS ONE.

[108] Zhang A., Li M., Wang B., Klein J.D., Price S.R., Wang X.H. (2018). miRNA-23a/27a attenuates muscle atrophy and renal fibrosis through muscle-kidney crosstalk. J. Cachexia Sarcopenia Muscle.

[109] Mogi M., Kohara K., Nakaoka H., Kan-No H., Tsukuda K., Wang X.L., Chisaka T., Bai H.Y., Shan B.S., Kukida M. (2016). Diabetic mice exhibited a peculiar alteration in body composition with exaggerated ectopic fat deposition after muscle injury due to anomalous cell differentiation. J. Cachexia Sarcopenia Muscle.

[110] Tahamtan M., Moosavi S.M., Sheibani V., Nayebpour M., Esmaeili-Mahani S., Shabani M. (2016). Erythropoietin attenuates motor impairments induced by bilateral renal ischemia/reperfusion in rats. Fundam. Clin. Pharmacol..

[111] Workeneh B.T., Mitch W.E. (2010). Review of muscle wasting associated with chronic kidney disease. Am. J. Clin. Nutr..

[112] Mimura I., Nangaku M., Nishi H., Inagi R., Tanaka T., Fujita T. (2010). Cytoglobin, a novel globin, plays an antifibrotic role in the kidney. Am. J. Physiol. Renal. Physiol..

[113] Fujii C., Miyashita K., Mitsuishi M., Sato M., Fujii K., Inoue H., Hagiwara A., Endo S., Uto A., Ryuzaki M. (2017). Treatment of sarcopenia and glucose intolerance through mitochondrial activation by 5-aminolevulinic acid. Sci. Rep..

[114] Tamaki M., Hagiwara A., Miyashita K., Wakino S., Inoue H., Fujii K., Fujii C., Sato M., Mitsuishi M., Muraki A. (2015). Improvement of Physical Decline Through Combined Effects of Muscle Enhancement and Mitochondrial Activation by a Gastric Hormone Ghrelin in Male 5/6Nx CKD Model Mice. Endocrinology.

[115] Nakamura S., Yoshimori T. (2017). New insights into autophagosome-lysosome fusion. J. Cell. Sci..

[116] Lorincz P., Juhasz G. (2020). Autophagosome-Lysosome Fusion. J. Mol. Biol..

[117] Sandri M. (2013). Protein breakdown in muscle wasting: Role of autophagy-lysosome and ubiquitin-proteasome. Int. J. Biochem. Cell. Biol..

[118] Wang D.T., Yang Y.J., Huang R.H., Zhang Z.H., Lin X. (2015). Myostatin Activates the Ubiquitin-Proteasome and Autophagy-Lysosome Systems Contributing to Muscle Wasting in Chronic Kidney Disease. Oxid. Med. Cell. Longev..

[119] Yokozawa T., Zheng P.D., Oura H. (1984). Biochemical features induced by adenine feeding in rats. Polyuria, electrolyte disorders, and 2,8-dihydroxyadenine deposits. J. Nutr. Sci. Vitaminol..

[120] Yokozawa T., Zheng P.D., Oura H., Koizumi F. (1986). Animal model of adenine-induced chronic renal failure in rats. Nephron.

[121] Diwan V., Small D., Kauter K., Gobe G.C., Brown L. (2014). Gender differences in adenine-induced chronic kidney disease and cardiovascular complications in rats. Am. J. Physiol. Renal. Physiol..

[122] Yang C., Liu C., Zhou Q., Xie Y.C., Qiu X.M., Feng X. (2015). Effect of atracylodes rhizome polysaccharide in rats with adenine-induced chronic renal failure. Indian J. Pharm. Sci..

[123] Vanholder R., Schepers E., Pletinck A., Nagler E.V., Glorieux G. (2014). The uremic toxicity of indoxyl sulfate and p-cresyl sulfate: A systematic review. J. Am. Soc. Nephrol..

[124] Changchien C.Y., Lin Y.H., Cheng Y.C., Chang H.H., Peng Y.S., Chen Y. (2019). Indoxyl sulfate induces myotube atrophy by ROS-ERK and JNK-MAFbx cascades. Chem. Biol. Interact..

[125] Moe S.M., Chen N.X., Seifert M.F., Sinders R.M., Duan D., Chen X., Liang Y., Radcliff J.S., White K.E., Gattone V.H. (2009). A rat model of chronic kidney disease-mineral bone disorder. Kidney Int..

[126] Umegaki H. (2015). Sarcopenia and diabetes: Hyperglycemia is a risk factor for age-associated muscle mass and functional reduction. J. Diabetes Investig..

[127] Carlson M.E., Suetta C., Conboy M.J., Aagaard P., Mackey A., Kjaer M., Conboy I. (2009). Molecular aging and rejuvenation of human muscle stem cells. EMBO Mol. Med..

[128] Lee C.E., McArdle A., Griffiths R.D. (2007). The role of hormones, cytokines and heat shock proteins during age-related muscle loss. Clin. Nutr..

[129] Garry G.A., Antony M.L., Garry D.J. (2016). Cardiotoxin Induced Injury and Skeletal Muscle Regeneration. Methods Mol. Biol..

[130] Fujimaki S., Seko D., Kitajima Y., Yoshioka K., Tsuchiya Y., Masuda S., Ono Y. (2018). Notch1 and Notch2 Coordinately Regulate Stem Cell Function in the Quiescent and Activated States of Muscle Satellite Cells. Stem. Cells..

[131] Nishi H., Inagi R., Kawada N., Yoshizato K., Mimura I., Fujita T., Nangaku M. (2011). Cytoglobin, a novel member of the globin family, protects kidney fibroblasts against oxidative stress under ischemic conditions. Am. J. Pathol..

[132] Fu Y., Tang C., Cai J., Chen G., Zhang D., Dong Z. (2018). Rodent models of AKI-CKD transition. Am. J. Physiol. Renal. Physiol..

[133] Wei Q., Dong Z. (2012). Mouse model of ischemic acute kidney injury: Technical notes and tricks. Am. J. Physiol. Renal. Physiol..

[134] Zhang Y., Nakano D., Guan Y., Hitomi H., Uemura A., Masaki T., Kobara H., Sugaya T., Nishiyama A. (2018). A sodium-glucose cotransporter 2 inhibitor attenuates renal capillary injury and fibrosis by a vascular endothelial growth factor-dependent pathway after renal injury in mice. Kidney Int..

[135] Guan Y., Nakano D., Zhang Y., Li L., Tian Y., Nishiyama A. (2019). A mouse model of renal fibrosis to overcome the technical variability in ischaemia/reperfusion injury among operators. Sci. Rep..

[136] Osberg J.W., Meares G.J., McKee D.C., Burnett G.B. (1982). Intellectual functioning in renal failure and chronic dialysis. J. Chronic. Dis..

[137] Castaneda C., Gordon P.L., Uhlin K.L., Levey A.S., Kehayias J.J., Dwyer J.T., Fielding R.A., Roubenoff R., Singh M.F. (2001). Resistance training to counteract the catabolism of a low-protein diet in patients with chronic renal insufficiency. A randomized, controlled trial. Ann. Intern. Med..

[138] Inagi R. (2010). Endoplasmic reticulum stress as a progression factor for kidney injury. Curr. Opin. Pharmacol..

[139] Inagi R., Ishimoto Y., Nangaku M. (2014). Proteostasis in endoplasmic reticulum--new mechanisms in kidney disease. Nat. Rev. Nephrol..

[140] Maekawa H., Inagi R. (2019). Pathophysiological Role of Organelle Stress/Crosstalk in AKI-to-CKD Transition. Semin. Nephrol..

[141] Jheng J.R., Chen Y.S., Ao U.I., Chan D.C., Huang J.W., Hung K.Y., Tarng D.C., Chiang C.K. (2018). The double-edged sword of endoplasmic reticulum stress in uremic sarcopenia through myogenesis perturbation. J. Cachexia Sarcopenia Muscle.

[142] Frigeri A., Nicchia G.P., Verbavatz J.M., Valenti G., Svelto M. (1998). Expression of aquaporin-4 in fast-twitch fibers of mammalian skeletal Muscle. J. Clin. Investig..

[143] Yoshimura K., Sugiura K., Ohmori Y., Aste N., Saito N. (2011). Immunolocalization of aquaporin-4 in the brain, kidney, skeletal muscle, and gastro-intestinal tract of chicken. Cell. Tissue. Res..

[144] Basco D., Blaauw B., Pisani F., Sparaneo A., Nicchia G.P., Mola M.G., Reggiani C., Svelto M., Frigeri A. (2013). AQP4-dependent water transport plays a functional role in exercise-induced skeletal muscle adaptations. PLoS ONE.

[145] Lin I.H., Chang J.L., Hua K., Huang W.C., Hsu M.T., Chen Y.F. (2018). Skeletal muscle in aged mice reveals extensive transformation of muscle gene expression. BMC Genet..

[146] Gamboa J.L., Billings F.T., Bojanowski M.T., Gilliam L.A., Yu C., Roshanravan B., Roberts L.J., Himmelfarb J., Ikizler T.A., Brown N.J. (2016). Mitochondrial dysfunction and oxidative stress in patients with chronic kidney disease. Physiol. Rep..

[147] Grobet L., Martin L.J., Poncelet D., Pirottin D., Brouwers B., Riquet J., Schoeberlein A., Dunner S., Ménissier F., Massabanda J. (1997). A deletion in the bovine myostatin gene causes the double-muscled phenotype in cattle. Nat. Genet..

[148] McPherron A.C., Lawler A.M., Lee S.J. (1997). Regulation of skeletal muscle mass in mice by a new TGF-beta superfamily member. Nature.

[149] Verzola D., Barisione C., Picciotto D., Garibotto G., Koppe L. (2019). Emerging role of myostatin and its inhibition in the setting of chronic kidney disease. Kidney Int..

[150] Cash J.N., Rejon C.A., McPherron A.C., Bernard D.J., Thompson T.B. (2009). The structure of myostatin:follistatin 288: Insights into receptor utilization and heparin binding. EMBO J..

[151] Schafer M.J., Atkinson E.J., Vanderboom P.M., Kotajarvi B., White T.A., Moore M.M., Bruce C.J., Greason K.L., Suri R.M., Khosla S. (2016). Quantification of GDF11 and Myostatin in Human Aging and Cardiovascular Disease. Cell. Metab..

[152] Becker C., Lord S.R., Studenski S.A., Warden S.J., Fielding R.A., Recknor C.P., Hochberg M.C., Ferrari S.L., Blain H., Binder E.F. (2015). Myostatin antibody (LY2495655) in older weak fallers: A proof-of-concept, randomised, phase 2 trial. Lancet Diabetes Endocrinol..

[153] Woodhouse L., Gandhi R., Warden S.J., Poiraudeau S., Myers S.L., Benson C.T., Hu L., Ahmad Q.I., Linnemeier P., Gomez E.V. (2016). A Phase 2 Randomized Study Investigating the Efficacy and Safety of Myostatin Antibody LY2495655 versus Placebo in Patients Undergoing Elective Total Hip Arthroplasty. J. Frailty Aging.

[154] Rooks D., Praestgaard J., Hariry S., Laurent D., Petricoul O., Perry R.G., Lach-Trifilieff E. (2017). Treatment of Sarcopenia with Bimagrumab: Results from a Phase, I.I.; Randomized, Controlled, Proof-of-Concept Study. J. Am. Geriatr. Soc..

[155] Pradhan G., Samson S.L., Sun Y. (2013). Ghrelin: Much more than a hunger hormone. Curr. Opin. Clin. Nutr. Metab. Care..

[156] Currow D.C., Abernethy A.P. (2014). Anamorelin hydrochloride in the treatment of cancer anorexia-cachexia syndrome. Future Oncol..

[157] Garcia J.M., Polvino W.J. (2009). Pharmacodynamic hormonal effects of anamorelin, a novel oral ghrelin mimetic and growth hormone secretagogue in healthy volunteers. Growth Horm IGF Res..

[158] Garcia J.M., Boccia R.V., Graham C.D., Yan Y., Duus E.M., Allen S., Friend J. (2015). Anamorelin for patients with cancer cachexia: An integrated analysis of two phase 2, randomised, placebo-controlled, double-blind trials. Lancet Oncol..

[159] Nishikawa M., Ishimori N., Takada S., Saito A., Kadoguchi T., Furihata T., Fukushima A., Matsushima S., Yokota T., Kinugawa S. (2015). AST-120 ameliorates lowered exercise capacity and mitochondrial biogenesis in the skeletal muscle from mice with chronic kidney disease via reducing oxidative stress. Nephrol. Dial. Trans..

[160] Ishikawa I., Araya M., Hayama T., Sugano M., Yamato H., Ise M. (2002). Effect of oral adsorbent (AST-120) on renal function, acquired renal cysts and aortic calcification in rats with adriamycin nephropathy. Nephron.

[161] Owada S., Maeba T., Sugano Y., Hirayama A., Ueda A., Nagase S., Goto S., Nishijima F., Bannai K., Yamato H. (2010). Spherical carbon adsorbent (AST-120) protects deterioration of renal function in chronic kidney disease rats through inhibition of reactive oxygen species production from mitochondria and reduction of serum lipid peroxidation. Nephron. Exp. Nephrol..

[162] Akizawa T., Asano Y., Morita S., Wakita T., Onishi Y., Fukuhara S., Gejyo F., Matsuo S., Yorioka N., Kurokawa K. (2009). Effect of a carbonaceous oral adsorbent on the progression of CKD: A multicenter, randomized, controlled trial. Am. J. Kidney Dis..

[163] Schulman G., Berl T., Beck G.J., Remuzzi G., Ritz E., Arita K., Kato A., Shimizu M. (2015). Randomized Placebo-Controlled EPPIC Trials of AST-120 in CKD. J. Am. Soc. Nephrol..

[164] Schulman G., Berl T., Beck G.J., Remuzzi G., Ritz E., Shimizu M., Shobu Y., Kikuchi M. (2016). The effects of AST-120 on chronic kidney disease progression in the United States of America: A post hoc subgroup analysis of randomized controlled trials. BMC Nephrol..

[165] Inoue R., Nishi H., Tanaka T., Nangaku M. (2019). Regional variance in patterns of prescriptions for chronic kidney disease in Japan. Clin. Exp. Nephrol..

[166] Chan M.C., Holt-Martyn J.P., Schofield C.J., Ratcliffe P.J. (2016). Pharmacological targeting of the HIF hydroxylases—A new field in medicine development. Mol. Aspects Med..

[167] Jelkmann W. (2007). Erythropoietin after a century of research: Younger than ever. Eur. J. Haematol..

[168] Luberg K., Wong J., Weickert C.S., Timmusk T. (2010). Human TrkB gene: Novel alternative transcripts, protein isoforms and expression pattern in the prefrontal cerebral cortex during postnatal development. J. Neurochem..

[169] Buemi M., Cavallaro E., Floccari F., Sturiale A., Aloisi C., Trimarchi M., Corica F., Frisina N. (2003). The pleiotropic effects of erythropoietin in the central nervous system. J. Neuropathol. Exp. Neurol..

[170] Nairz M., Sonnweber T., Schroll A., Theurl I., Weiss G. (2012). The pleiotropic effects of erythropoietin in infection and inflammation. Microbes Infect..

[171] Sanghani N.S., Haase V.H. (2019). Hypoxia-Inducible Factor Activators in Renal Anemia: Current Clinical Experience. Adv. Chronic. Kidney Dis..

[172] Hasegawa S., Jao T.M., Inagi R. (2017). Dietary Metabolites and Chronic Kidney Disease. Nutrients.

[173] Anusornvongchai T., Nangaku M., Jao T.M., Wu C.H., Ishimoto Y., Maekawa H., Tanaka T., Shimizu A., Yamamoto M., Suzuki N. (2018). Palmitate deranges erythropoietin production via transcription factor ATF4 activation of unfolded protein response. Kidney Int..

[174] Jao T.M., Nangaku M., Wu C.H., Sugahara M., Saito H., Maekawa H., Ishimoto Y., Aoe M., Inoue T., Tanaka T. (2019). ATF6alpha downregulation of PPARalpha promotes lipotoxicity-induced tubulointerstitial fibrosis. Kidney Int..

[175] Nishi H., Nangaku M. (2019). Podocyte lipotoxicity in diabetic kidney disease. Kidney Int..

[176] Ertunc M.E., Hotamisligil G.S. (2016). Lipid signaling and lipotoxicity in metaflammation: Indications for metabolic disease pathogenesis and treatment. J. Lipid Res..

[177] Han J., Kaufman R.J. (2016). The role of ER stress in lipid metabolism and lipotoxicity. J. Lipid Res..

[178] Nishi H., Higashihara T., Inagi R. (2019). Lipotoxicity in Kidney, Heart, and Skeletal Muscle Dysfunction. Nutrients.

[179] Hammond S.M., Wood M.J. (2011). Genetic therapies for RNA mis-splicing diseases. Trends Genet..

